# Effects of Carbon Content and Current Density on the Li^+^ Storage Performance for MnO@C Nanocomposite Derived from Mn-Based Complexes

**DOI:** 10.3390/nano10091629

**Published:** 2020-08-19

**Authors:** Ranran Jiao, Li Zhao, Shuli Zhou, Yanjun Zhai, Denghu Wei, Suyuan Zeng, Xianxi Zhang

**Affiliations:** School of Chemistry and Chemical Engineering, Liaocheng University, Liaocheng 252059, China; JRR1175@163.com (R.J.); zhaoli1175@163.com (L.Z.); zhouli1996@126.com (S.Z.); zhaiyanjun@lcu.edu.cn (Y.Z.); weidenghu@lcu.edu.cn (D.W.)

**Keywords:** MnO@C nanocomposites, carbon content, current density, lithium-ion battery, transition metal oxide anode

## Abstract

In this study, a simple method was adopted for the synthesis of MnO@C nanocomposites by combining in-situ reduction and carbonization of the Mn_3_O_4_ precursor. The carbon content, which was controlled by altering the annealing time in the C_2_H_2_/Ar atmosphere, was proved to have great influences on the electrochemical performances of the samples. The relationships between the carbon contents and electrochemical performances of the samples were systematically investigated using the cyclic voltammetry (CV) as well as the electrochemical impedance spectroscopy (EIS) method. The results clearly indicated that the carbon content could influence the electrochemical performances of the samples by altering the Li^+^ diffusion rate, electrical conductivity, polarization, and the electrochemical mechanism. When being used as the anode materials in lithium-ion batteries, the capacity retention rate of the resulting MnO@C after 300 cycles could reach 94% (593 mAh g^−1^, the specific energy of 182 mWh g^−1^) under a current density of 1.0 A g^−1^ (1.32 C charge/discharge rate). Meanwhile, this method could be easily scaled up, making the rational design and large-scale application of MnO@C possible.

## 1. Introduction

Over the past years, rechargeable lithium-ion batteries (LIBs) have attracted ever-growing attention in energy-related fields, such as portable electronic devices and electrical vehicles, because of its long lifespan, environmental benignity, high energy density, and low memory effect [1,2,3]. Although graphite is widely used as commercial anode materials, the low specific capacity (372 mAh g^−1^) and poor rate performance of graphite can’t meet the ever-increasing requirement [4]. Thus, it’s of the urgent need to explore alternative anode materials with excellent specific capacity as well as long cycling stability.

Transition metal oxides have been considered to be promising candidate anode materials for LIBs due to its advantages, such as high theoretical capacity, low toxicity, environment friendliness, and affordable cost [5,6,7]. Among them, manganese monoxide (MnO) is particularly attractive because of its high theoretical capacity as well as low voltage hysteresis [8,9]. By a conversion process based on MnO + 2Li^+^ + e^−^ → Mn + Li_2_O, MnO can deliver a theoretical discharge capacity of 755.6 mAh g^−1^, making it a good candidate as the anode material in LIBs. However, the large-scale application of MnO as the anode material in LIBs is still not applicable. One of the difficulties is the poor cycling stability, which is mainly ascribed to the large volume change during the charge/discharge process due to the precipitation of Li_2_O. The other difficulty is the low electrical conductivity of MnO, which will inevitably restrict its power performance [10]. To solve these problems, the enhancement of the mechanical strength, as well as the electrical conductivity of the MnO, is of vital importance. To date, the most commonly employed method is the so-called carbon coating method, which combines MnO with carbonaceous materials, such as graphite and graphene. The incorporation between MnO and carbonaceous materials can improve the electrical conductivity, as well as the mechanical strength of MnO, rendering it improved electrochemical performances. As a result, a large number of composite materials based on MnO and carbonaceous materials have been successfully prepared [11,12,13]. For example, Cheng and co-workers reported the fabrication of MnO nanoparticles within a 3D graphene-based carbon network, which demonstrated superior electrochemical performance as the anode for LIBs [14]. The capacity retention rate can reach 100% after cycling 600 times, while the specific capacity of pure MnO decreases to ~25% after just 30 cycles. Guo and co-workers also synthesized MnO@C nanocomposites, which could deliver a discharge capacity of 421 mAh g^−1^ at a current density of 100 mA g^−1^ [15]. Compared to pure MnO, both the discharge capacity and cycling stability are greatly improved. Although great achievements have been made, most of these reports concern on the fabrication of the composite structures. The detailed influence of carbon content is still not clear, which makes the rational design of MnO@C composite materials difficult to achieve.

**Figure 1:**
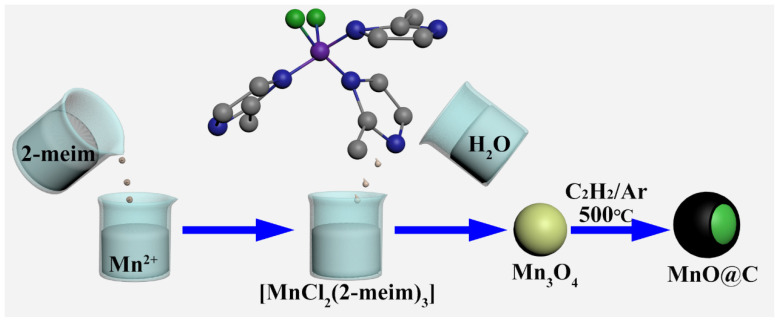
Schematic illustration for the preparation of MnO@C nanocomposite.

In this work, a simple method incorporating hydrolysis, as well as in-situ carbonization, was adopted for the synthesis of MnO@C nanocomposite (Figure 1). By controlling the annealing time in the C_2_H_2_/Ar atmosphere, the carbon contents in the samples could be easily controlled. Obvious differences in electrochemical performances could be clearly observed among the samples with different carbon contents. Sample with the best performance could be obtained by annealing the precursor at 500 °C for 180 min, which could deliver a discharge capacity of 593 mAh g^−1^ (specific energy of 182 mWh g^−1^) after 300 cycles under the current density of 1.0 A g^−1^ (1.32 C charge/discharge rate). The capacity retention rate could still reach 94% after 300 cycles, indicating high stability during the charge-discharge cycles. To reveal the relationship between carbon contents and electrochemical performances, both cyclic voltammetry (CV) and electrochemical impedance spectroscopy (EIS) methods were employed. The corresponding results clearly indicated that the carbon contents could influence the electrochemical behaviors of the samples by altering the Li^+^ diffusion rate, electrical conductivity, polarization, and the electrochemical mechanism during the charge-discharge process. The final electrochemical performance could be regarded as the comprehensive result of these effects mentioned above. Meanwhile, this method could be easily scaled up by simply enlarging the amount of raw materials, making it a candidate method for the preparation of MnO@C nanocomposites.

## 2. Materials and Methods

### 2.1. Chemicals

All chemicals were of analytical grade and used without further purification.

### 2.2. Synthesis of the [MnCl_2_(2-meim)_3_] Complexes and MnO@C Nanoparticles

The preparation of [MnCl_2_(2-meim)_3_] was carried out according to the previous report with some modifications [16]. The schematic illustration of the synthetic process is shown in Figure 1. In a typical experiment, 2.2 g manganese chloride tetrahydrate (MnCl_2_·4H_2_O, Aladdin, Shanghai, China) was dissolved in 40 mL absolute methanol (Aladdin, Shanghai, China) to form solution A. At the same time, 4.0 g 2-Methylimidazole (2-meim, Aladdin, Shanghai, China) was dissolved in another 40 mL absolute methanol to form solution B. In the next step, solution B was poured into solution A under vigorous stirring. The mixed solution was stirred for another 20 min to obtain the brown [MnCl_2_(2-meim)_3_] solution. Finally, 80 mL de-ionized water was poured into the above [MnCl_2_(2-meim)_3_] solution and stirred for 1 h to ensure the total transformation from [MnCl_2_(2-meim)_3_] to Mn_3_O_4_ nanoparticles. The MnO@C nanocomposites were obtained by annealing the above-mentioned Mn_3_O_4_ nanoparticles under a C_2_H_2_/Ar (10 vol% C_2_H_2_) atmosphere at 500 °C for different times. The samples obtained by annealing for 80 min, 180 min, and 360 min were labeled as MC-80, MC-180, and MC-360, respectively.

### 2.3. Materials Characterization

X-ray powder diffraction (XRD) patterns of the samples were recorded on a diffractometer (Rigaku Smartlab 9, Tokyo, Japan) with Cu K_α_ radiation (*λ* = 1.5406 Å). Electron microscopy characterizations of the samples were conducted on HRTEM (high-resolution transmission electron microscopy, Thermo Fischer, Talos F200x, Waltham, MA, USA) with an accelerating voltage of 200 kV. Raman spectra were measured using an Invia Raman microscope (Invia Microscope, Renishaw, Wotton-under-Edge, Gloucestershire, UK). X-ray photoelectron spectra of the samples were conducted using ESCALAB 250 instrument (Thermo Fischer, EACALAB 250, Waltham, MA, USA). The surface areas and pore size distributions of the samples were acquired by the nitrogen adsorption/desorption apparatus (Quantachrome autosorb IQ-C, Graz, Steiermark, Austria).

### 2.4. Electrochemical Measurement

Electrochemical Measurements were tested using CR2032 coin-type half cells (Hefei Kejing technology Co., Ltd., Anhui, China). The working electrode was prepared by ball-milling active materials, super-P carbon (Hefei Kejing technology Co., Ltd, Anhui, China), and sodium carboxymethyl cellulose (CMC, Aladdin, Shanghai, China) binder (7:2:1, wt%) to form a homogeneous slurry with copper as a current collector and then dried in a vacuum at 90 °C for 12 h. The loading of the active materials was about 1.0 mg cm^−2^. The lithium disk served as both a reference and counter electrode, and the Celgard 2400 film (Hefei Kejing technology Co., Ltd, Anhui, China) was used as a separator. LiPF_6_ dissolved in ethylene carbonate/diethyl carbonate (EC/DEC) (1:1 in volume, 1 mol L^−1^) with fluoroethylene carbonate (FEC, 5% in weight) additive agent was used as the electrolyte. The assembly process was completed in a glovebox filled with an argon atmosphere (H_2_O < 0.1 ppm, O_2_ < 0.1 ppm). The constant current charge and discharge performance were tested between 0.01 and 3.0 V using the LAND CT2001A system (Wuhan LAND Electronics Co., Ltd., Wuhan, China) at room temperature. All the assembled cells were tested at the current density of 0.1 A g^−1^ to activate for the first three cycles. Cyclic voltammetry (CV) plots were recorded from 0.01 to 3.0 V using GAMRY reference 600+ electrochemical workstation (Gamry Instruments, Philadelphia, PA, USA), and the electrochemical impedance spectroscopy (EIS) plots were obtained from 0.01 Hz to 100 kHz using the same instrument.

## 3. Results and Discussions

### 3.1. Composition and Microstructures of MnO@C Nanocomposites

The MnO@C nanocomposites were fabricated by a simple hydrolysis process, followed by calcination of the precursor under C_2_H_2_/Ar (10 vol% C_2_H_2_) atmosphere. In the first step, manganese chloride tetrahydrate (MnCl_2_·4H_2_O) coordinated with 2-Methylimidazole (2-meim) and led to the formation of a brown solution, indicating the formation of a complex between Mn (II) and 2-meim. By evaporating the solvent, the residual solid was determined to be MnCl_2_(2-meim)_3_, according to the XRD examination (Supporting information, Figure S1). For this complex, a Cl atom and a 2-meim ligand occupied the axial position, with Mn–Cl and Mn–N distance being determined to be 2.525 and 2.249 Å, respectively. For the equatorial groups, the respective bonds were somewhat shorter—a Cl at 2.392 Å and two 2-meim ligands with a mean Mn–N of 2.195 Å [16]. In the next step, the above-mentioned brown solution hydrolyzed upon the addition of water and resulted in the formation of Mn_3_O_4_ nanoparticles. The formation of Mn_3_O_4_ nanoparticles could be clearly verified by the corresponding XRD patterns (Supporting Information, Figure S2). All the diffraction peaks could be indexed to be the tetragonal phased Mn_3_O_4_ (JCPDS, Card No. 01-075-1560). It should be noted that this reaction can be easily scaled up by simply enlarging the amount of starting material, which makes it a promising route for the large-scale synthesis of Mn_3_O_4_ nanoparticles (Supporting Information, Figure S3). The detailed structure of Mn_3_O_4_ was further investigated by HRTEM observation (Supporting Information, Figure S4). According to the HRTEM observation, the as-obtained Mn_3_O_4_ was composed of nanoparticles with a size of ~25 nm. Meanwhile, the EDS mapping analysis confirmed the homogeneous distribution of elements Mn and O. These nanoparticles were poly-crystallized according to the ring-like SAED (selected area electron diffraction) patterns, which could be indexed to be the tetragonal phase Mn_3_O_4_. The lattice spacing, being determined to be 0.276 nm and 0.288 nm, corresponded to the (1 0 3) and (2 0 0) planes of tetragonal phased Mn_3_O_4_. The angles between the (1 0 3) and (2 0 0) lattice planes were measured to be 61°, which was consistent with the theoretical value of tetragonal Mn_3_O_4_. When the as-obtained Mn_3_O_4_ was annealed in the C_2_H_2_/Ar atmosphere, simultaneous carbonization, as well as reduction, occurred, leading to the formation of MnO@C composite structures.

**Figure 2:**
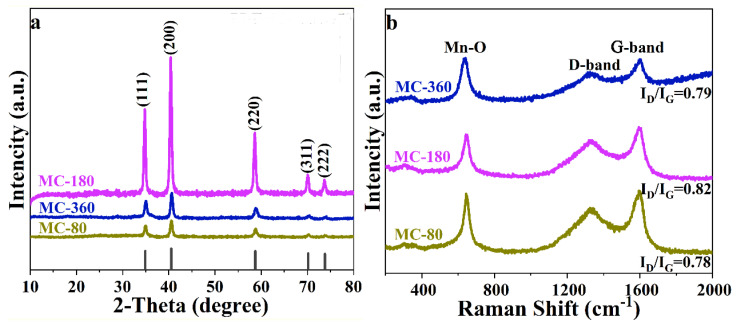
(**a**) XRD patterns and (**b**) Raman spectra of MC-80, MC-180, and MC-360.

The phase purities of the as-prepared samples were examined using the power X-ray powder diffraction (XRD), and the corresponding result is shown in Figure 2a. For all the three samples, the diffraction peaks centering at 34.8°, 40.4°, 58.6°, 70.1°, 73.7° could be designated to the (1 1 1), (2 0 0), (2 2 0), (3 1 1), (2 2 2) crystal planes of cubic phase MnO (JCPDS Card NO. 01-077-2363). No other diffraction peak was detected, indicating the high purity of the samples. The diffraction peak of carbon was also not detected, which might result from the low diffraction intensity of carbon. To verify the existence of carbon in the as-prepared three samples, Raman spectra were employed, and the results are shown in Figure 2b. The peak centering at 647 cm^−1^ could be ascribed to the vibration of Mn–O bands [17]. The obvious peaks centering at 1332 and 1594 cm^−1^ could be ascribed to the D and G bands of carbon, which clearly indicated the existence of carbon in these three samples [18]. The intensity ratio between the D and G peak (I_D_/I_G_) could be used to evaluate the graphitization degrees of the as-prepared samples [19,20]. The calculated values for samples MC-80, MnC-180, MC-360 were determined to be 0.78, 0.82, and 0.79, indicating that the three samples were partially graphitized [21].

**Figure 3:**
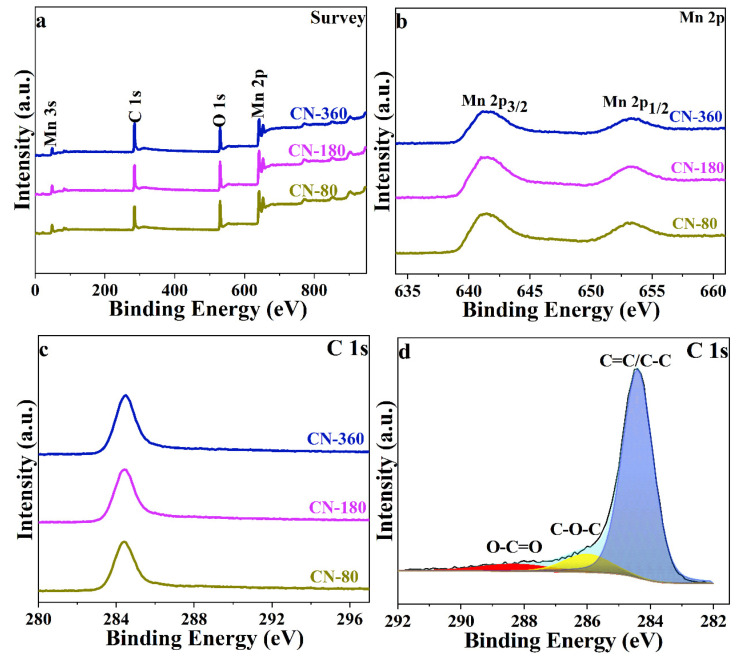
XPS survey spectra (**a**), Mn 2p spectra (**b**), C 1s spectra (**c**) of MC-80, MC-180, and MC-360, and C 1s spectrum of MC-180 (**d**).

The detailed information on elemental compositions and the valence states were further investigated using the X-ray photoelectron spectroscopy (XPS). As it is shown in Figure 3a, all three samples were composed of Mn, O, and C, according to the overall survey spectra. Figure 3b shows the Mn 2p spectra for the three samples, on which two apparent peaks locating at 641.6 and 653.5 eV could be assigned to Mn 2p_3/2_ and Mn 2p_1/2_, respectively. The separation between Mn 2p_3/2_ and Mn 2p_1/2_ was 11.9 eV, which was in agreement with the previous reports for MnO [22,23]. In the C 1s spectrum, all the three samples had similar XPS spectra (Figure 3c), and the deconvoluted energy bands could be assigned to C=C/C–C (284.4 eV), C–O–C (286.0 eV), and O–C=O (288.3 eV) (Figure 3d) [24].

**Figure 4:**
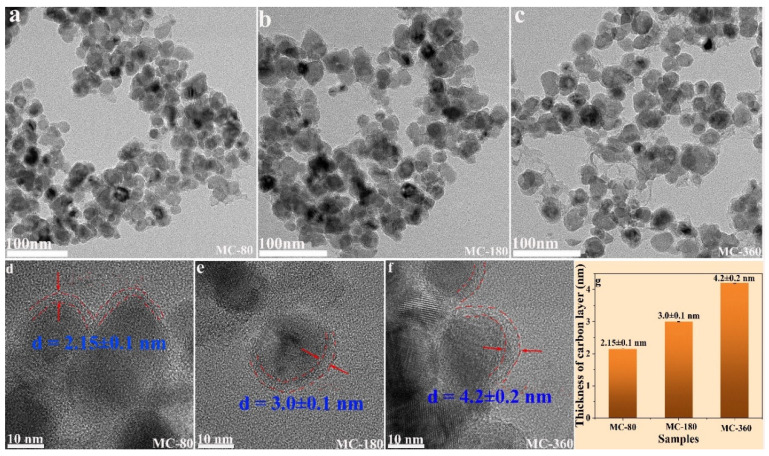
TEM (**a**–**c**) and HRTEM (**d**–**f**) images of samples MC-80, M-180, and MC-360. Histogram of the carbon thickness of samples MC-80, M-180, and MC-360 (**g**).

The microstructural, as well as the morphological features for the three samples (MC-80, MC-180, MC-360), were further investigated using HRTEM (Figure 4). For all the three samples, the size of the nanoparticles slightly increased as compared to the Mn_3_O_4_ precursor. A carbon layer could be clearly observed on the surfaces of MnO nanoparticles, indicating the formation of MnO@C composite structures. The thickness of the carbon layer, being determined from the HRTEM images, was 2.15 ± 0.1 nm, 3 ± 0.1 nm, and 4.2 ± 0.2 nm for samples MC-80, MC-180, and MC-360, respectively. Obviously, the thickness of the carbon layer became thicker as the annealing time increased.

**Figure 5:**
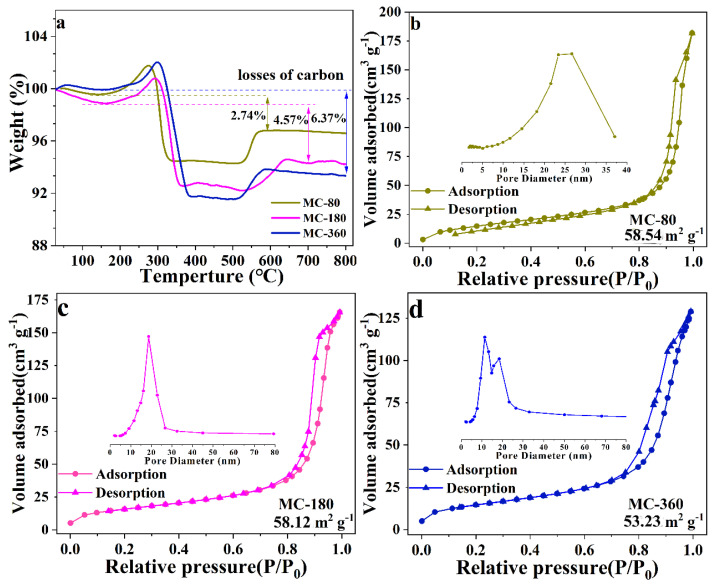
(**a**) TG (thermogravimetry) curves of MC-80, MC-180, and MC-360 (atmosphere: air, heating rate: 10 °C min^−1^). (**b**–**d**) N_2_ adsorption-desorption isotherms of MC-80, MC-180, and MC-360, and the inset figures are corresponding size distribution curves.

To find out the exact carbon contents of the three samples, thermogravimetry (TG) was employed, and the corresponding results are shown in Figure 5a. All three samples showed mass losses before 170 °C, which might result from the loss of adsorbed water in the air. As the temperature increased further, a small weight could be observed, which might result from the partial oxidation of MnO [8]. The residual carbon could be totally removed in the temperature range from 300 °C to 400 °C, which would lead to significant weight losses on the TG curves. In the range between 500 °C and 600 °C, an evident weight increase could be ascribed to the further oxidation of MnO to Mn_2_O_3_ [9,25]. This hypothesis could be verified by the XRD examination of the samples calcined at 700 °C in air, which could be designated to be Mn_2_O_3_, according to the XRD pattern (Supporting information, Figure S5). Based on the transformation from MnO@C to Mn_2_O_3_, the carbon contents for the as-obtained three samples MC-80, MC-180, and MC-360 were calculated to be 12.58, 14.23, and 15.85%, respectively.

The porous natures of samples MC-80, MC-180, and MC-360 were further examined using the nitrogen isothermal adsorption-desorption method (Figure 5b–d). For all three samples, the adsorption-desorption curves could be designated to be type Ⅳ with an evident H1 type hysteresis loop, indicating that all three samples possessed interconnected pores with narrow size distribution [26]. The Brunauer–Emmert–Teller (BET) surface area of samples MC-80, MC-180, and MC-360 were determined to be 58.54 m^2^ g^−1^, 58.12 m^2^ g^−1^, and 53.23 m^2^ g^−1^, respectively. Large specific surface areas could enhance the contact between electrolyte and active materials, which would be beneficial to improve the electrochemical properties of the active materials. The pore size distribution curves (inset of Figure 5b–d) obtained using the Barrett–Joyner–Halenda (BJH) method revealed a mesopore size distribution on the basis of IUPAC classification. The pore sizes of MC-80 and MC-180 mainly concentrated on 25 and 18.9 nm, respectively. The pore sizes of MC-360 mainly concentrated on 11.4 and 18.4 nm.

### 3.2. Electrochemical Property in Half-Cells

**Figure 6:**
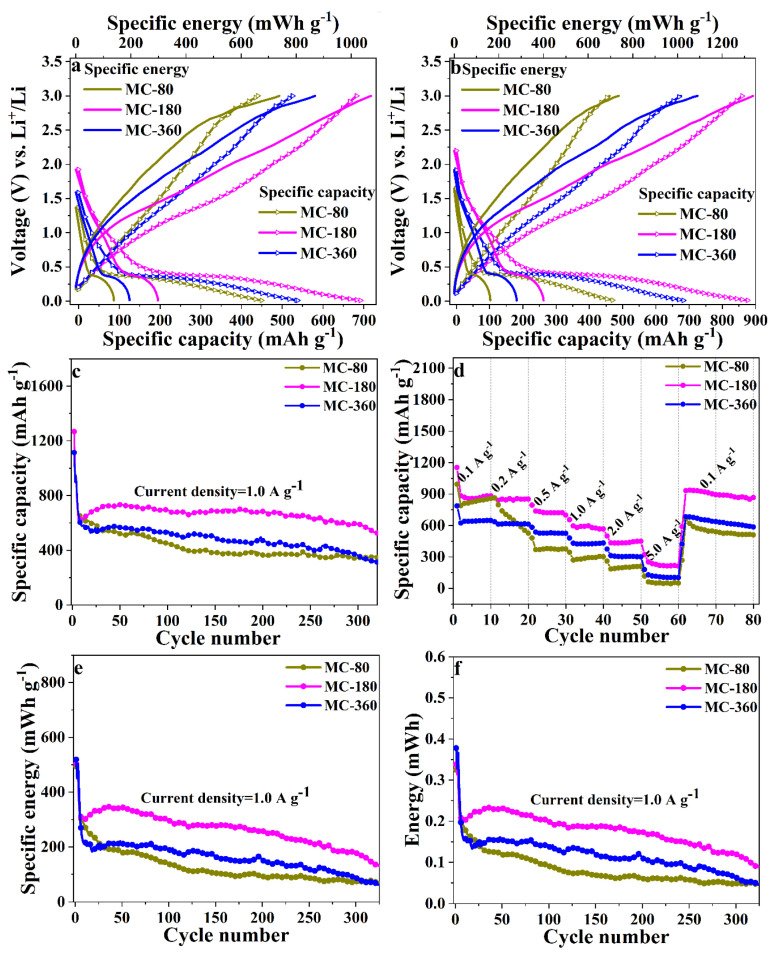
The charge-discharge voltage profiles at the current density of 1.0 A g^−1^ (**a**) and 0.5 A g^−1^ (**b**) for the 100th cycle. Cycle performance (**c**) at the current density 1.0 A g^−1^, and the rate performance (**d**) of MC-80, MC-180, and MC-360. The specific energy (**e**) and absolute energy (**f**) of MC-80, MC-180, and MC-360 at the current density of 1.0 A g^−1^.

To evaluate the electrochemical performances of the as-obtained three samples, galvanostatic charge and discharge profiles were tested within the voltage window of 0.01–3.0 V (current density of 1.0 and 0.5 A g^−1^, 1.32 and 0.66 C charge/discharge rate). During the initial charge/discharge cycle, sample MC-80 possessed a high initial Coulombic efficiency of 72.7%, which was related to the formation of solid electrolyte interface (SEI) layer and/or the drastic Li-driven structural changes. The initial Coulombic efficiencies for samples MC-180 and MC-360 were 70.3 and 69.6%, respectively, resulting from the irreversible capacities of carbon shells (Supplementary Information, Figure S6). For all the three samples, a long voltage plateau at 0.25 V could be observed in the initial discharge process, which was attributed to the reduction of Mn^2+^ to Mn^0^ (Supplementary Information, Figure S7). As for the subsequent discharge processes, the voltage plateaus moved to 0.4 V, indicating the irreversible formation of crystalline metal nanoparticles and amorphous Li_2_O matrixes. During the subsequent charge processes, the sloped plateaus between 1.25 and 2.0 V were related to the oxidation of Mn^0^ to Mn^2+^ [10]. The discharge voltage plateau was still around 0.4 V after 100 cycles, indicating the reversible charge/discharge process (Figure 6a,b). After 100 cycles, the discharge specific capacity (specific energy) for the samples MC-80, MC-180, and MC-360 under a current density of 1 A g^−1^ were determined to be 470 (159), 877 (400), and 685 (282) mAh g^−1^ (mWh g^−1^). To further investigate the electrochemical performances of the three samples, long-cycle stability tests of the samples were also carried out (Figure 6c). Among the three samples, sample MC-180 showed the best performance as compared to the other two samples. The reversible discharge capacities of MC-180 were 683 mAh g^−1^ and 590 mAh g^−1^ after cycling for 150 and 300 times, with a capacity retention rate of about 94% after 300 cycles. While for the other two samples MC-80 and MC-360, the capacity retention rates were 53% and 61% after 300 cycles, respectively. Obviously, sample MC-180 exhibited the highest cycling stability among the three samples. For comparison purpose, the galvanostatic charge and discharge profile of MnO nanoparticles without carbon coating was also tested (Supplementary Information, Figure S8). The discharge capacity rapidly faded to less than 100 mAh g^−1^ after 20 cycles, indicating the poor cycling stability of bare MnO nanoparticles. To facilitate the comparison with other electrochemical systems, the changes in specific energy and absolute energy during the charge/discharge cycles were also plotted (Figure 6e,f). The rate performances of the three samples were also investigated under different current densities (0.1–5 A g^−1^, 0.13–6.6 C charge/discharge rate), and the corresponding results are shown in Figure 6d. The average specific capacities of sample MC-180 under the current density of 0.1, 0.2, 0.5, 1.0, 2.0, and 5.0 A g^−1^ were determined to be 856, 846, 722, 584, 433, and 216 mAh g^−1^, respectively. As the current density returned to 0.1 A g^−1^, a specific capacity of 884 mAh g^−1^ was recovered, which was higher than the corresponding values in the initial 10 cycles. This behavior has been widely reported for the transitional metal oxides, which can be mainly ascribed to the generation of higher oxidation states and the activation process [27,28]. These experimental results clearly indicate that the carbon contents of the samples do have a great influence on the electrochemical performances of the samples. Although the exact reason for the differences is still not clear, the deduced reasons could be described from two aspects. First, excessive coating of carbon will restrain the permeation of electrolyte and exert a barrier on the inward/outward Li^+^ ion, which will be harmful to the electrochemical performance of the samples. Secondly, an excess of carbon would pull down the specific capacity.

### 3.3. Electrochemical Mechanism of the MnO@C Nanocomposites

**Figure 7:**
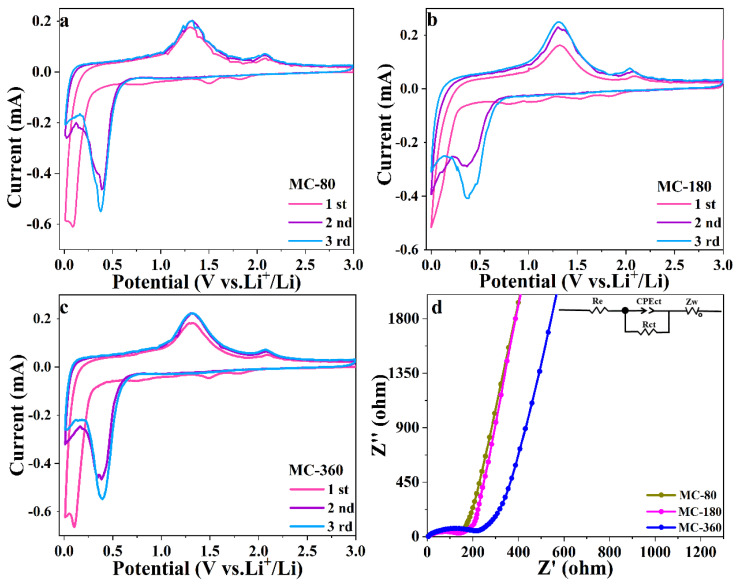
CV (cyclic voltammetry) curves of MC-80 (**a**), MC-180 (**b**), and MC-360 (**c**) at a scan rate of 0.1 mV s^−1^. (**d**) Nyquist plots of fresh cells for MC-80, MC-180, and MC-360, and the inset is the equivalent circuit.

The electrochemical performances of MnO-based materials reported in recent works were also compared, and the corresponding results are summarized in Table S1. Although the carbon thicknesses of the MnO nanoparticles were adjustable, the electrochemical performances of the samples were not satisfying. To get further insight into the electrochemical behavior of the three samples, a series of experiments was conducted. In the first step, the cyclic voltammetry (CV) method was employed to examine the electrochemical mechanism during the charge-discharge process (Figure 7). All three samples exhibited similar electrochemical behaviors according to the CV curves. As for the initial cathodic progress, a series of reduction peaks during 1.4–1.8 V could be ascribed to the partial reduction of oxidized Mn (II) and the formation of solid electrolyte interphase (SEI) layer. These weak peaks would disappear during the subsequent cycles, indicating the irreversible formation of SEI film [29]. The sharp peak below 0.1 V could be attributed to the reduction of MnO to metallic Mn (MnO + 2Li^+^ + 2e^+^ → Mn + Li_2_O) [30]. In the subsequent cycles, the reduction peak slightly moved to 0.38 V, which could be assigned to the activation process [31]. While for the anodic process, the peak centering at 1.32 V could be ascribed to the oxidation process from Mn to MnO (Mn + Li_2_O → MnO + 2Li^+^ + 2e^+^) [32]. What is more, the peak centering at 2.09 V gradually appeared as the cycle number increased, which could be assigned to further oxidization of Mn^2+^ [28]. It is worth noting that the CV curves remained consistent for the second and third cycles, indicating the good electrochemical reversibility of the three samples [33,34].

Besides the CV method, EIS measurement was also conducted, and the corresponding results are shown in Figure 7d. Although the three samples underwent a similar electrochemical transformation during the charge-discharge process, the EIS spectra did indicate obvious differences among the three samples. For all the three samples, the Nyquist plots of fresh cells could be divided into two parts—one semicircle in the high-frequency region and one slope line in the low-frequency region. The corresponding equivalent circuit model is shown as the inset of Figure 7d, and the fitting results are shown in Table S2 (Supporting Information, Table S2). The detailed values of charge transfer resistance (R_ct_) for samples MC-80, MC-180, and MC-360 were determined to be 150.9, 113.3, and 163.5 Ω, respectively. Obviously, sample MC-180 had the smallest charge transfer resistance among the three samples, indicating the enhanced electrochemical kinetics behavior for sample MC-180. The slope in the low-frequency region corresponded to the diffusion resistance of Li^+^. Sample MC-180 had the largest line slope as compared to samples MC-80 and MC-360, indicating the fastest Li^+^ diffusion rate. It is generally accepted that the carbon coating could improve inter-particle conductivity, especially for particles with a size of less than 40 nm [35]. As for the three samples in our experiments, the carbon contents of the samples gradually increased with the annealing time. However, the electrochemical performances didn’t always increase as the function of carbon content. The increase of carbon content would be beneficial to the electrochemical performance as the carbon content increased from 12.58% (MC-80) to 14.23% (MC-180). While the carbon contents increased from 14.23 to 15.85%, the electrochemical performance of the sample was greatly weakened. Although carbon coating is a crucial factor in improving the electrical conductivity and mechanical properties of the active materials, it doesn’t mean that a thick carbon shell is required [35].

**Figure 8:**
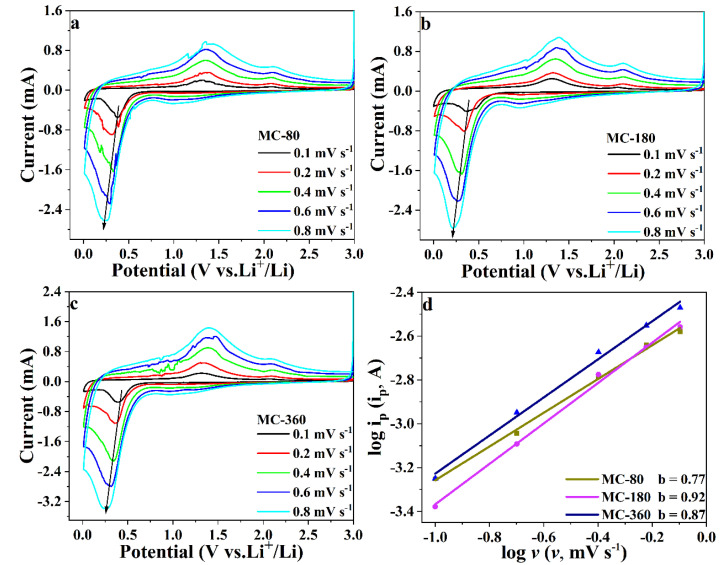
CV curves of MC-80 (**a**), MC-180 (**b**), and MC-360 (**c**) at different scan rates, and the relationship between the log (*i*) and log (*v*) to determine the *b*-values (**d**).

To further understand the influence of carbon content, the capacitive effects of the three samples were investigated by conducting CV tests at different sweep rates (Figure 8a–c). For the description of the electrochemical process, three mechanisms were mainly employed [36]. The first one was the non-faradaic contribution from the double-layer capacitance, while the second one was the so-called faradaic behavior owing to lithium-ion insertion and extraction. The last one was based on the pseudocapacitive behavior arising from surface faradaic redox reaction. The detailed mechanism of the electrochemical process could be distinguished using the equations below,

*i* = *av^b^* (1)

log *i* = *b* log *v* + log *a* (2)

where *a* and *b* are adjustable coefficients, *v* and *i* are scan rate and peak current during the CV process [37,38]. The value of *b* represents the charge storage kinetics of the electrode and can be calculated from the slope of Equation (2). When *b* = 1, the charge transfer process is an ideal capacitive process, referring to the fast kinetics of the electrode [39]. While when *b* = 0.5, it belongs to a diffusion-controlled process [40]. In our experiments, the values of *b* were calculated from reduction peaks, and the corresponding results are shown in Figure 8d. The value of *b* for sample MC-180 was about 0.922, suggesting that the discharge process was pseudocapacitive. As for samples MC-80 and MC-360, the values of *b* were determined to be 0.77 and 0.87, respectively, which were smaller than the corresponding value of sample MC-180. This result clearly indicated that the diffusion-controlled process was more dominant for samples MC-80 and MC-360, especially for sample MC-80. According to the previous reports, pseudocapacitive charge storage represents the faradaic charge-transfer reaction, including fast solid ion intercalations and surface redox reaction, which is beneficial to the long-term cyclability and rate performance [41]. This could be the reason why sample MC-180 possessed the best electrochemical performances among the three samples.

**Figure 9:**
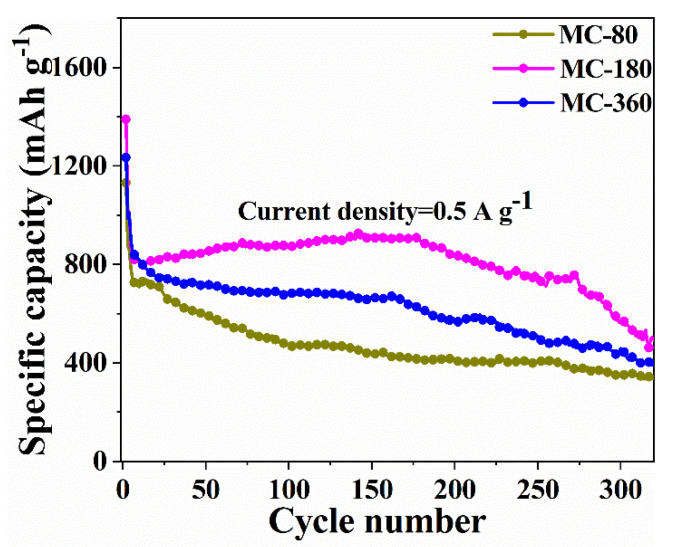
Cycle performance of samples MC-80, MC-180, and MC-360 at the current density 0.5 A g^−1^.

To verify this hypothesis, the charge-discharge processes were also conducted under a current density of 0.5 A g^−1^ (0.66 C), and the corresponding results are shown in Figure 9. The capacity retention rates of MC-180 under the current density of 0.5 A g^−1^ and 1.0 A g^−1^ after 150 cycles were 111 and 107%, respectively (Supplementary Information, Table S3). However, the capacity retention rate after 300 cycles was only 71%, which was much lower than the corresponding value under the current density of 1.0 A g^−1^ (94%). Meanwhile, MC-80 could only deliver a discharge capacity of 435 mAh g^−1^ and 351 mAh g^−1^ after 150 and 300 cycles under the current density of 0.5 A g^−1^, which were also much lower than the corresponding value under the current density of 1 A g^−1^. The capacity retention rates for all the three samples after 150 cycles and 300 cycles at the current of 0.5 A g^−1^ are shown in Table S3, revealing inferior electrochemical performances under low current density.

**Figure 10:**
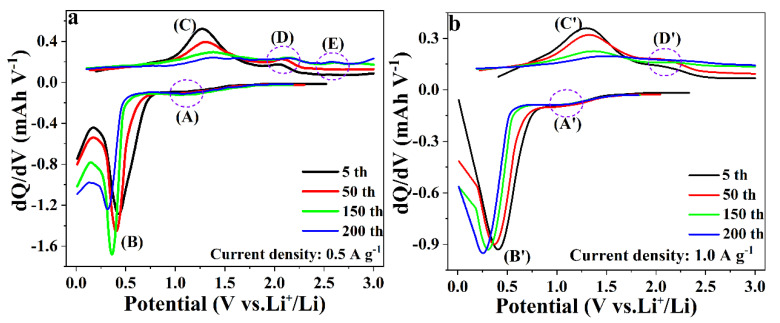
Differential capacities (dQ/dV) versus voltage plots of the charge and discharge curves for the current density 0.5 A g^−1^ (**a**) and 1.0 A g^−1^ (**b**) for MC-180.

Obviously, the as-synthesized three samples showed superior electrochemical performances under high current density. Wang and co-workers speculated that this phenomenon might be related to the complete and slow Li-ion reactions at a smaller current, which would lead to more serious electrode pulverization [42]. Lou and co-workers ascribed it to a sufficient reaction at a low current rate, which would lead to more severe volume changes and side reactions [43]. In order to examine the differences between large and small current density, differential capacities, dQ/dV, were calculated and plotted against voltage (Figure 10). In this plot, peaks (B) and (B’) were designated to be a reduction of MnO to metallic Mn (at ~0.41 V), which corresponded to the sloped plateaus in the range of 0.35–0.51 V for the discharge profile. (C) and (C’) were assigned to the oxidation process from Mn to MnO, which corresponded to the sloped plateaus in the range of 1.31–1.55 V for the charge profiles. The other pairs of small peaks (A and D, A’ and D’ in Figure 10) were assigned to the oxidation of Mn^2+^ to higher oxidation states and the reduction of Mn^3+^/Mn^4+^ to Mn^2+^, respectively. As it is shown in Figure 10, the peaks for the discharge curves gradually moved toward low voltage upon the increasing of cycles. On the contrary, the peaks for the charge curves moved toward higher voltage. This phenomenon could be ascribed to the enhanced polarization during the charge and discharge process. When the charge-discharge cycles reached to 150 under the current density of 0.5 A g^−1^, a new peak (E) centering at the ~2.58 V appeared. However, there was no corresponding reduction peak on the discharge curve, indicating that some irreversible reactions occurred. When the current density was 1.0 A g^−1^, no extra peak appeared (Figure 10b). It could be concluded that a low current rate could lead to a more irreversible reaction. The experimental results clearly indicated that the side reactions during the charge-discharge process were the key point for the inferior electrochemical performance under low current density.

**Figure 11:**
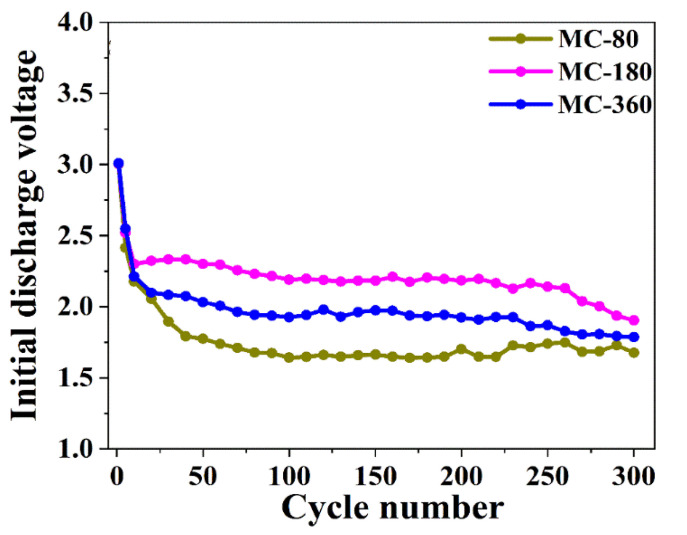
The initial discharge voltage at every cycle of MC-80, MC-180, and MC-360.

In the experiment, the galvanostatic charge and discharge tests were also carried out within the voltage window between 0.01 and 3.0 V. However, the true end-of-charge voltage was not 3.0 V because of the existence of polarization at the electrolyte-electrode interface. The polarization caused by a local imbalance between the cell reaction and ion diffusion might lead to an accelerated capacity fading during the charge-discharge process. The initial discharge voltage for all the three samples at the first cycle was about 3.0 V, which was consistent with the open-circuit voltage of the cell (Figure 11). However, the initial discharge voltage at every cycle gradually decreased along with the increase of charge-discharge cycles. As it is shown in Figure 11, sample MC-180 had the largest initial discharge voltage. The initial discharge voltage at every cycle among the three samples followed the sequence as MC-180 > MC-360 > MC-80. The larger initial discharge voltage indicated that the electrode materials had a smaller polarization and enhanced electrochemistry performance. For the three samples, sample MC-80 had low inter-particle conductivity due to the low carbon content, which would increase the polarization. Although the carbon content of MC-360 was higher than MC-180, excess carbon shell would hinder the penetration of the electrolyte into the active materials, which also increased the polarization. Thus, the polarization of the electrode was the synergistic effect of the two factors mentioned above.

## 4. Conclusions

In summary, we developed a simple method for the synthesis of MnO@C nanocomposites with different carbon contents. Sample with the best performance could be obtained by annealing the precursor in C_2_H_2_/Ar for 180 min at 500 °C. Even after cycling for 300 times, it could still deliver a discharge capacity 593 mAh g^−1^ (specific energy of 182 mWh g^−1^), with a capacity retention rate of 94%. Meanwhile, the as-synthesized sample showed excellent rate performance, which could be ascribed to the pseudocapacitive process during cycles. By influencing the Li^+^ diffusion rate, electrical conductivity, polarization, and electrochemical mechanism, the electrochemical performances of the samples could be greatly influenced by the carbon contents.

## Supplementary Materials

The following are available online at https://www.mdpi.com/2079-4991/10/9/1629/s1, Figure S1: XRD patterns of this work and simulated [MnCl_2_(2-meim)_3_]; Figure S2: XRD pattern of Mn_3_O_4_ nanoparticles; Figure S3: Mass-products of brown [MnCl_2_(2-meim)_3_] solution; Figure S4: (**a**) TEM image, (**b**) HRTEM image, (**c**) SAED pattern, (**d**) HADDF image, and (**e**–**f**) elemental mapping results of the as-obtained Mn_3_O_4_; Figure S5: XRD pattern of MC-180 after annealing at 700 °C under air atmosphere; Figure S6: The Coulombic efficiency and energy efficiency of MC-80, MC-180, and MC-360 at the current density of 0.5 A g^−1^ (**a**) and 1.0 A g^−1^ (**b**), and the specific energy (**c**) and absolute energy (**d**) of MC-80, MC-180, and MC-360 at the current density of 0.5 A g^−1^; : The charge-discharge voltage profiles at the current density of 1.0 A g^−1^ (**a**–**c**) and 0.5 A g^−1^ (**d**–**f**) for MC-80, MC-180, and MC-360; Figure S8: The galvanostatic charge and discharge profiles and Coulombic efficiency of MnO nanoparticles without carbon coating at the current density of 0.5 A g^−1^; Table S1: Comparison of cycling performance for this work with reported MnO-based anode materials recently; Table S2: Simulated EIS parameters of MC-80, MC-180, and MC-360; Table S3: Capacity retention rates after 150 and 300 cycles at 0.5 A g^−1^ and 1.0 A g^−1^.
