# Supplementary material for: Effects of Carbon Content and Current Density on the Li+ Storage Performance for MnO@C Nanocomposite Derived from Mn-Based Complexes
Source: Nanomaterials (Basel). 2020 Aug 19;10(9):1629. doi: 10.3390/nano10091629 (PMC7560032; doi:10.3390/nano10091629)
Supplement: Supplementary file 1 [file nanomaterials-10-01629-s001.pdf]

nanomaterials

Article

Effects of Carbon Content and Current Density on the Li⁺ Storage Performance for MnO@C Nanocomposite Derived from Mn-Based Complexes

Ranran Jiao [1], Li Zhao [1], Shuli Zhou [1], Yanjun Zhai [1], Denghu Wei [1], Suyuan Zeng [1,*], and Xianxi Zhang [1,*]

[1] School of Chemistry and Chemical Engineering, Liaocheng University, 252059 Liaocheng, China; JRR1175@163.com (R.J.); 3236842769@qq.com (L.Z.); 1037600098@qq.com (S.Z.); zhaiyanjun@lcu.edu.cn (Y.Z.); weidenghu@lcu.edu.cn (D.W.)

* Correspondence: drzengsy@163.com(S.Z.); xxzhang3@126.com(X.Z.); Tel: +86-635-8230614, Fax: +86-635-8230196

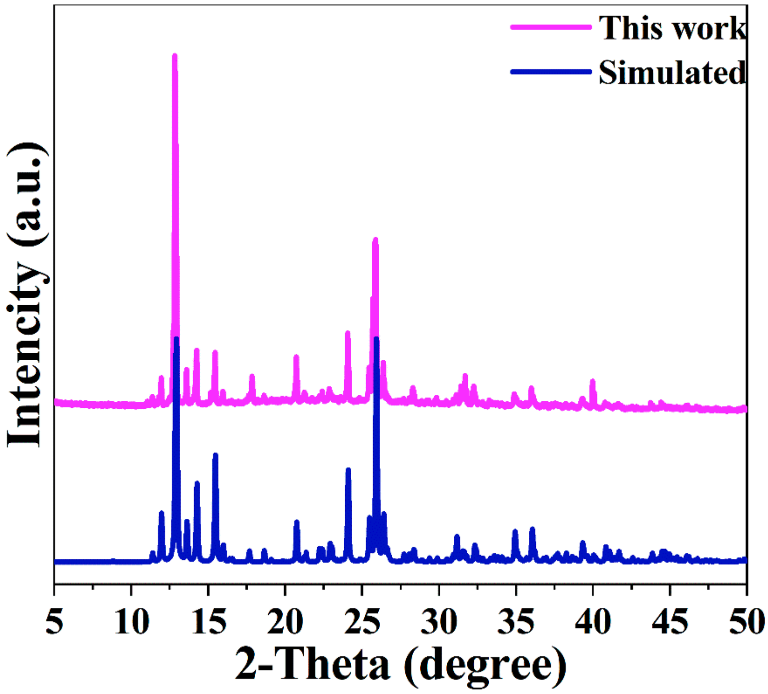

Figure 1. XRD patterns of this work and simulated [$MnCl_2$(2-meim)$_3$]. We obtained the solid samples by vaporizing absolute methanol at room temperature for several days.

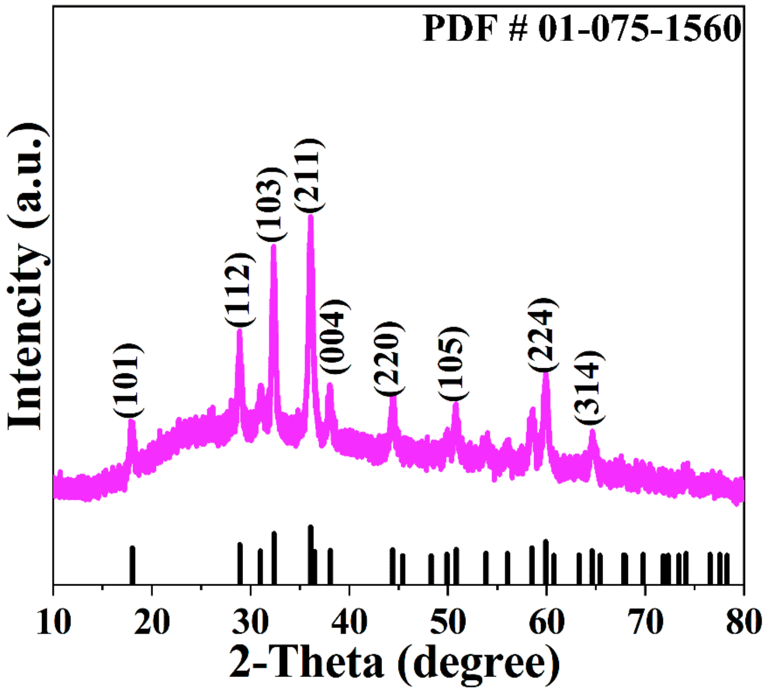

Figure 2. XRD pattern of Mn_3O_4 nanoparticles.

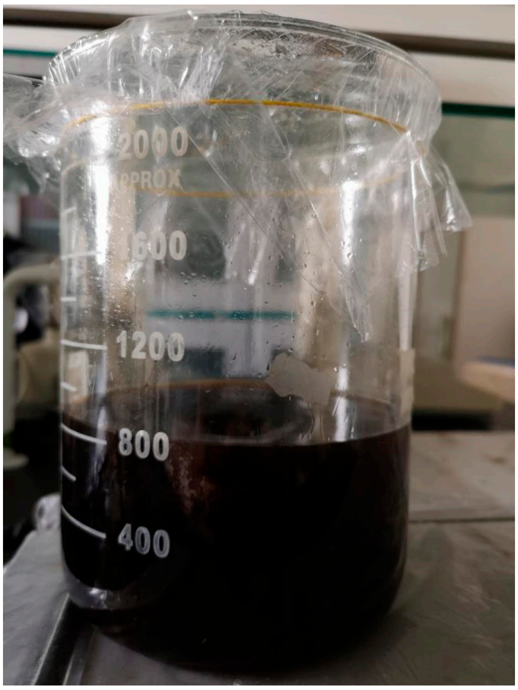

Figure 3. Mass-products of brown $[MnCl_2(2\text{-meim})_3]$ solution.

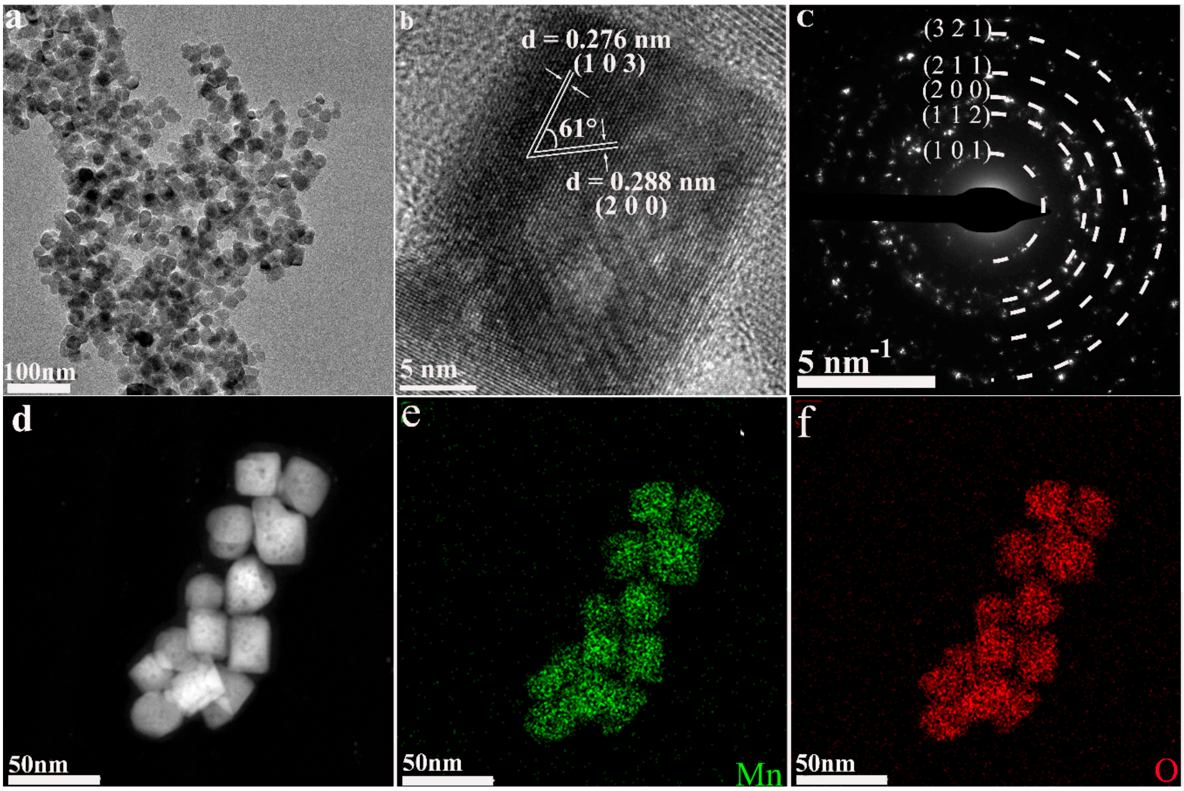

Figure 4. (**a**) TEM image, (**b**) HRTEM image, (**c**) SAED pattern (**d**) HADDF image and (**e, f**) elemental mapping results of the as-obtained Mn_3O_4.

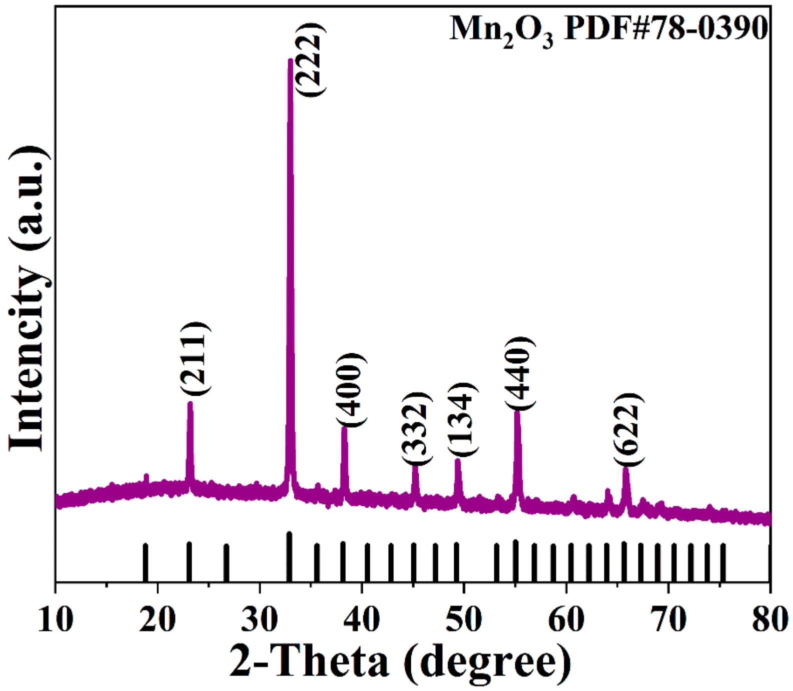

Figure 5. XRD pattern of MC-180 after annealing at 700 °C under air atmosphere.

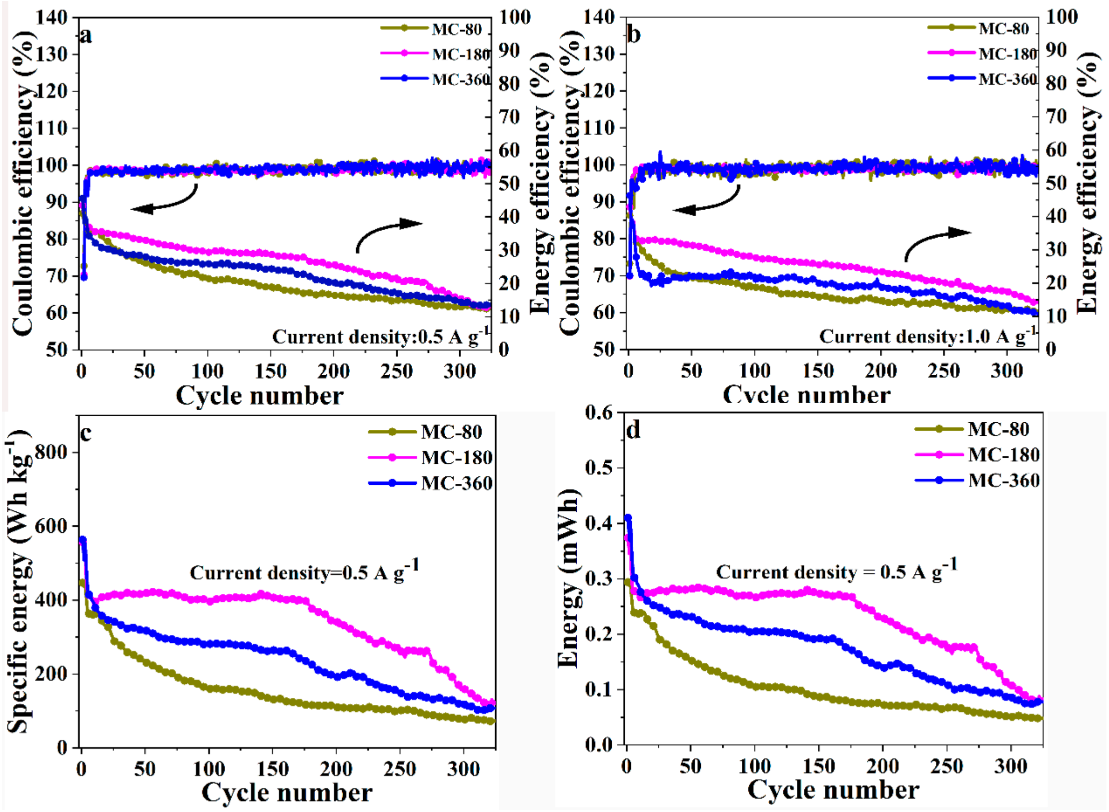

Figure 6. The Coulombic efficiencies and energy efficiencies of MC-80, MC-180 and MC-360 at the current density of 0.5 A g^{-1} (**a**) and 1.0 A g^{-1} (**b**). The specific energy (**c**) and absolute energy (**d**) of MC-80, MC-180 and MC-360 at the current density of 0.5 A g^{-1}.

Among the three samples, sample MC-180 shows the best performance as compared to the other two samples. The specific energies of MC-180 are 158 and 182 mWh g^{-1} after cycling for 300 times at the current density of 0.5 and 1.0 A g^{-1}, respectively. While for the other two samples MC-80 and MC-360, the specific energy are 79 and 116 mWh g^{-1} after 300 cycles at the current density of 0.5 A g^{-1}, respectively.

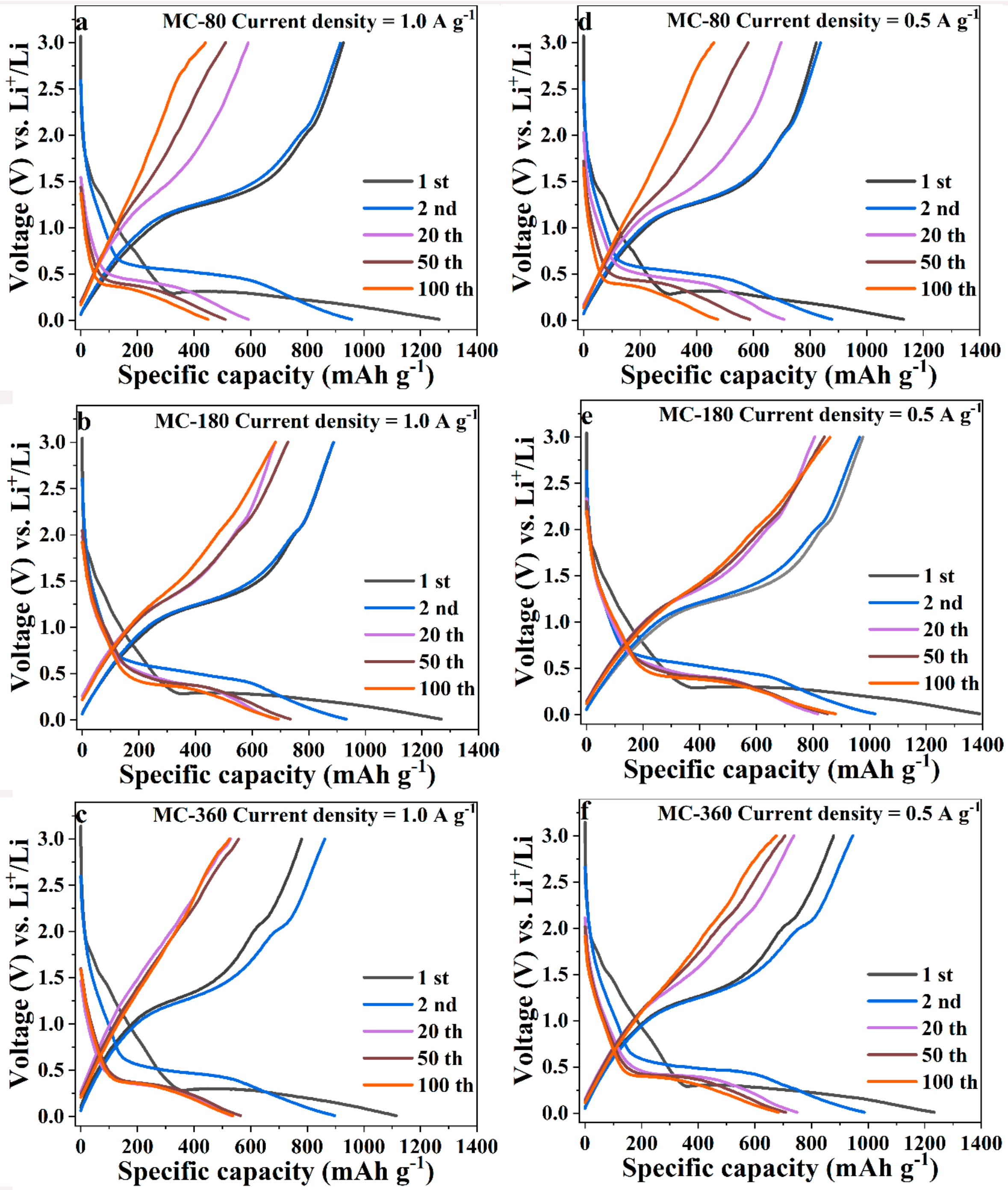

Figure 7. The charge-discharge voltage profiles at the current density of 1.0 A g^{-1} (**a–c**) and 0.5 A g^{-1} (**d–f**) for MC-80, MC-180 and MC-360.

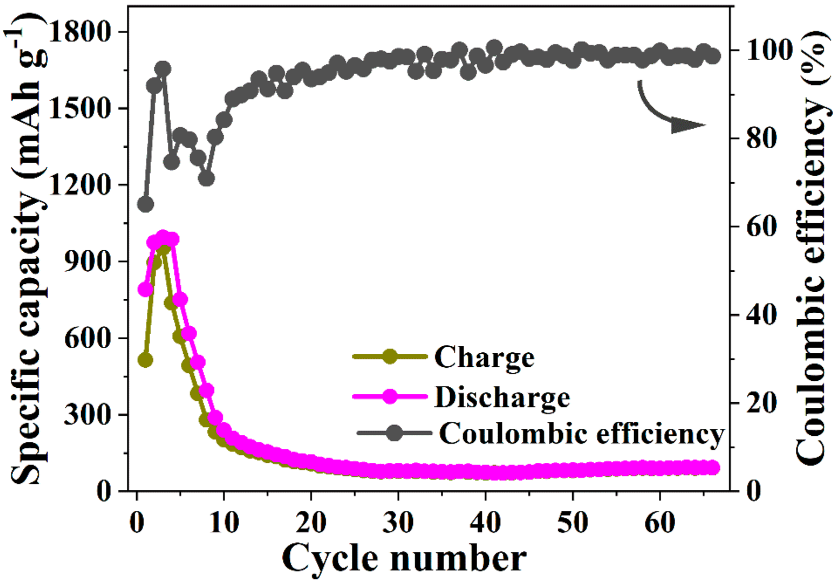

Figure 8. The galvanostatic charge and discharge profiles and coulombic efficiency of MnO nanoparticles without carbon coating at the current density of 0.5 A g^{-1}.

Table 1. Comparison of cycling performances for this work with reported MnO-based anode materials recently.

Mno Anode Materials	Current Density [A g^{-1}]	Cycle Number	Reversible Capacity [mA h g^{-1}]	References
MnO/C/graphene	0.1	350	754.3	[1]
Cross-linked MnO	0.3	500	687	[2]
MnO nanoparticles	1.0	1000	549	[3]
MnO/C@rGO	1.0	500	909.1	[4]
MnO/C nanowires	0.1	100	832	[5]
MnO@C microcages	0.1	270	1450.5	[6]
C/MnO nanosheets	5.0	2000	1467	[7]
MnO/C-N hybrid	0.5	170	1699	[8]
MnO/carbon framwork	0.2	200	939	[9]
MnO/GO composite	0.075	100	936	[10]
**This work**	0.5 (1.0)	300	563 (593)	-

Table 2. Simulated EIS parameters of MC-80, MC-180 and MC-360.

Electrode Materials	R_s (Ω)	R_{ct} (Ω)
MC-80	2.616	150.9
MC-180	4.454	113.3
MC-360	3.819	163.5

Table 3. Capacity retention rates after 150 and 300 cycles at 0.5 and 1.0 A g^{-1}.

Samples	Capacity Retention Rate after 150 Cycles at 0.5 A g^{-1}	Capacity Retention Rate after 300 Cycles at 0.5 A g^{-1}	Capacity Retention Rate after 150 Cycles at 1.0 A g^{-1}	Capacity Retention Rate after 300 Cycles at 1.0 A g^{-1}
MC-80	61%	49%	60%	53%

MC-180	111%	71%	105%	94%
MC-360	79%	54%	83%	61%

Reference

1. Wang, Y.; Ding, X.; Wang, F.; Li, J.; Song, S.; Zhang, H. Nanoconfined nitrogen-doped carbon-coated MnO nanoparticles in graphene enabling high performance for lithium-ion batteries and oxygen reduction reaction. *Chem Sci* **2016**, *7*, 4284–4290, doi:10.1039/c5sc04668h.
2. Zhang, J.; Ren, T.; Nayaka, G.P.; Dong, P.; Duan, J.; Li, X.; Zhang, Y.; Wang, D. Design of polydopamine-encapsulation multiporous MnO cross-linked with polyacrylic acid binder for superior lithium ion battery anode. *J. Alloy. Compd.* **2019**, *783*, 341–348, doi:10.1016/j.jallcom.2018.12.356.
3. Huang, H.-W.; Fan, S.-S.; Dong, W.; Zou, W.; Yan, M.; Deng, Z.; Zheng, X.; Liu, J.; Wang, H.-E.; Chen, L., et al. Nitrogen-doped graphene in-situ modifying MnO nanoparticles for highly improved lithium storage. *Appl. Surf. Sci.* **2019**, *473*, 893–901, doi:10.1016/j.apsusc.2018.12.230.
4. Tian, X.-M.; Zhao, D.-L.; Meng, W.-J.; Han, X.-Y.; Yang, H.-X.; Duan, Y.-J.; Zhao, M. Highly porous MnO/C@rGO nanocomposite derived from Mn-BDC@rGO as high-performance anode material for lithium ion batteries. *J. Alloy. Compd.* **2019**, *792*, 487–495, doi:10.1016/j.jallcom.2019.04.027.
5. Wang, J.-G.; Zhang, C.; Jin, D.; Xie, K.; Wei, B. Synthesis of ultralong MnO/C coaxial nanowires as freestanding anodes for high-performance lithium ion batteries. *J. Mater. Chem. A* **2015**, *3*, 13699–13705, doi:10.1039/c5ta02440d.
6. Hou, C.; Tai, Z.; Zhao, L.; Zhai, Y.; Hou, Y.; Fan, Y.; Dang, F.; Wang, J.; Liu, H. High performance MnO@C microcages with a hierarchical structure and tunable carbon shell for efficient and durable lithium storage. *J. Mater. Chem. A* **2018**, *6*, 9723–9736, doi:10.1039/c8ta02863j.
7. Xiao, Y.; Cao, M. Carbon-Anchored MnO Nanosheets as an Anode for High-Rate and Long-Life Lithium-Ion Batteries. *Acs Appl Mater Interfaces* **2015**, *7*, 12840–12849, doi:10.1021/acsami.5b02171.
8. Xiao, Y.; Wang, X.; Wang, W.; Zhao, D.; Cao, M. Engineering hybrid between MnO and N-doped carbon to achieve exceptionally high capacity for lithium-ion battery anode. *Acs Appl Mater Interfaces* **2014**, *6*, 2051–2058, doi:10.1021/am405142p.
9. Wang, S.; Xing, Y.; Xu, H.; Zhang, S. MnO nanoparticles interdispersed in 3D porous carbon framework for high performance lithium-ion batteries. *Acs Appl Mater Interfaces* **2014**, *6*, 12713–12718, doi:10.1021/am5027055.
10. Petnikota, S.; Srikanth, V.V.S.S.; Nithyadharseni, P.; Reddy, M.V.; Adams, S.; Chowdari, B.V.R. Sustainable Graphenothermal Reduction Chemistry to Obtain MnO Nanonetwork Supported Exfoliated Graphene Oxide Composite and its Electrochemical Characteristics. *Acs Sustain. Chem. Eng.* **2015**, *3*, 3205–3213, doi:10.1021/acssuschemeng.5b00791.
